# Development and validation of a Spanish-language spatial release from masking task in a Mexican population

**DOI:** 10.1121/10.0016850

**Published:** 2023-01-18

**Authors:** E. Sebastian Lelo de Larrea-Mancera, Rodolfo Solís-Vivanco, Yolanda Sánchez-Jimenez, Laura Coco, Frederick J. Gallun, Aaron R. Seitz

**Affiliations:** 1 Department of Psychology, University of California, 900 University Avenue, Riverside, California 92507, USA; 2 Laboratory of Cognitive and Clinical Neurophysiology, Instituto Nacional de Neurología y Neurocirugía Manuel Velasco Suárez (INNNMVS), Avenue Insurgentes Sur 3877, La Fama, Tlalpan, Mexico City, CDMX 14269, Mexico; 3 Neuro-Otology Department, INNNMVS, Mexico City, Mexico; 4 Department of Otolaryngology, Oregon Health & Science University, Portland, Oregon 97239, USA

## Abstract

This study validates a new Spanish-language version of the Coordinate Response Measure (CRM) corpus using a well-established measure of spatial release from masking (SRM). Participants were 96 Spanish-speaking young adults without hearing complaints in Mexico City. To present the Spanish-language SRM test, we created new recordings of the CRM with Spanish-language Translations and updated the freely available app (PART; https://ucrbraingamecenter.github.io/PART_Utilities/) to present materials in Spanish. In addition to SRM, we collected baseline data on a battery of non-speech auditory assessments, including detection of frequency modulations, temporal gaps, and modulated broadband noise in the temporal, spectral, and spectrotemporal domains. Data demonstrate that the newly developed speech and non-speech tasks show similar reliability to an earlier report in English-speaking populations. This study demonstrates an approach by which auditory assessment for clinical and basic research can be extended to Spanish-speaking populations for whom testing platforms are not currently available.

## I. INTRODUCTION

In recent years, there has been an increased awareness of the importance of auditory health, aligned with a new understanding of hearing as a dynamic process influenced by multiple factors across the lifespan (see WHO, 2021; Haile *et al.*, 2021). At the same time, there have been advances in technology that have improved the accessibility of several tasks and stimuli to evaluate auditory function. Such state-of-the-art auditory assessment tools implemented in portable, commercially available technologies, such as iPads, tablets, and smart phones, could further advance basic auditory science research and complement clinical practice (see Gallun, 2020; Gates *et al.*, 2021). However, one important gap in the accessibility of such tools is the lack of availability of assessments for non-English-speaking populations. This, when combined with limited access to electronic devices and/or the internet in rural communities, leads to an overrepresentation of research in Western, educated, industrialized, rich, and democratic (WEIRD) populations. The current study is an example of how freely available tools can be used to facilitate data collection among diverse populations. Such tools will afford assessment of differences in auditory processing based on linguistic background or population, with the long-term goal of improving evidence-based practice and assessment for people in non-English-speaking communities. Such research is also relevant in the United States due to the large population of Spanish-speakers (an estimated 41 × 10^6^ people; https://data.census.gov).

Previously, our group has introduced Portable Adaptive Rapid Testing (PART), a free app (https://braingamecenter.ucr.edu/cognitive-perceptual-assessments) that enables researchers to collect laboratory-grade data (Gallun *et al.*, 2018) on a diverse set of auditory processing measures using consumer hardware (e.g., smartphone, tablet, computer with standard headphones). PART is a platform that contains a variety of auditory, visual, and cognitive assessments that allow flexibility both regarding the parameterization of the individual tasks (e.g., number of trials, adaptive procedures, instructions, language of presentation, etc.) as well as the ability to create custom batteries of tasks that are appropriate to the needs of a given experiment. In addition, PART has a companion app called BGC Science (https://apps.apple.com/dm/app/bgc-science/id1508696910) that is participant facing, where the user can download the app on their own device and enter a “server code” that configures their device for the assigned study. Particularly relevant to psychoacoustics research, PART contains a powerful sound engine that supports the generation of acoustical stimuli (e.g., filtering, noise generation, and generation of specialized sound-types). Additionally, PART provides visualization tools to analyze the stimuli generated and allows for download of any of the generated custom stimulation. Studies have shown that psychoacoustic data collected via PART are resilient to moderate noise levels similar to that commonly found in environmental settings (Lelo de Larrea-Mancera *et al.*, 2020). PART has even been shown to produce reliable data when tested using participant-owned devices and in home settings (Lelo de Larrea-Mancera *et al.*, 2022). Thus, PART represents a class of digital assessment tools that can be used to help researchers and clinicians gather large-scale datasets necessary to attain a better understanding of auditory processes. To our knowledge, PART is the only freely available, cross-platform tool (e.g., iOS, Android, Mac, Windows) that can generate a wide range of psychoacoustical stimuli and provide a variety of different task structures to facilitate research-grade remote testing of central auditory processes.

While the promise of PART for collecting data sets from much larger samples suggests that it can help to reduce the focus of hearing research on WEIRD populations, one limitation of the PART system is that, to date, the testing has only been accessible to English-speaking participants (Lelo de Larrea-Mancera *et al.*, 2020; Lelo de Larrea-Mancera *et al.*, 2022; Srinivasan *et al.*, 2020; Diedesch *et al.*, 2021). Expanding the linguistic diversity of accessible auditory assessment tools such as PART could have a significant impact that benefits both basic science and clinical research. This is important not only to expand the reach of research but also to build a solid base for the science. Diversity in data collection will better inform the reproducibility of measures and their expected generalization to other populations. For example, there are concerns that extant language-based auditory function assessments might not be valid for some non-native speakers with varying degrees of second language experience (see von Hapsburg and Peña, 2002; Cooke *et al.*, 2008; Hisagi *et al.*, 2022). Thus, developing auditory assessment tools that can be delivered in languages beyond English is an important step toward moving clinical and basic science beyond the typical samples under research and healthcare (Henrich *et al.*, 2010).

As a first step towards attaining more robust, reliable, and representative auditory research and clinical landscape, we “localized” the PART app along with a battery of assessments of potential clinical utility (see Hoover *et al.*, 2019; Lelo de Larrea-Mancera *et al.*, 2020) to Spanish. Localization (not to be confused with the psychoacoustical meaning of determining the location of a sound source) is a standard term in software development that refers to the process of adapting software materials to the language and culture of an end user. The materials were specifically localized for a Spanish-speaking population in Mexico City. However, we note that just like the English-language Coordinate Response Measure (CRM) corpus, and other tests in PART that are used across a wide range of English-speaking individuals, localization of PART into Spanish with a Spanish-language CRM makes these tests of central auditory processing available to the diverse population that comprehends the Spanish-language.

The current project focuses on validating a new Spanish-language version of the CRM corpus, using a well-established measure of SRM. The CRM was developed by Moore (1981) and was further described by Bolia *et al.* (2000) and Brungart (2001) as a rapid and representative means to test speech intelligibility. The localization of the CRM to Spanish involved a range of challenges that would not be present for tests involving pure tones or synthetic stimuli. Such tests mostly involve simple translations of instructions, but the adaptation of the speech corpuses involves not only the translation of individual words, but also matching word meanings, sentence structures, and prosody of speech. Such matching is required for effective measurement. Localization of the CRM and the associated SRM task to Spanish thus required translation and recording of a matrix of stimuli in a manner that ensured temporal and phonetic balance across all sentences.

The CRM has been used extensively to test different aspects of speech-on-speech masking (e.g., Brungart, 2001; Arbogast *et al.*, 2005; Marrone *et al.*, 2008; Best *et al.*, 2012; Oh *et al.*, 2021). Speech understanding in the presence of competing speech is thought to be one of the most important challenges to audition in ecological settings (Cherry, 1953; McDermott, 2009) and is the most common complaint among those with hearing loss (Kochkin, 2002; McCormack and Fortnum, 2013). Importantly, the CRM stimuli can be set up in masker competition scenarios where maskers are placed in simulated auditory space either spatially colocated with the target or with different degrees of spatial separation from it (Arbogast *et al.*, 2006; Marrone *et al.*, 2008; Best *et al.*, 2012; Gallun *et al.*, 2013; Jakien *et al.*, 2017; Oh *et al.*, 2021). This “release” from masking provides a measure of the improvement in speech understanding in competition that can be achieved by the auditory segregation of spatial cues and is sensitive to both the effects of aging and hearing loss (Gallun *et al.*, 2013; Srinivasan *et al.*, 2016).

Although the primary purpose of this study was to validate the Spanish-language version of the SRM task, we also localized the rest of the PART batteries to Spanish and collected data on a one-hour battery of tests, including detection of frequency modulations, temporal gaps, and modulated broadband noise in the temporal, spectral, and spectrotemporal domains. The entire battery of tests was self-administered via PART with instructions and graphical user interfaces provided in Spanish. Apart from the Spanish-language localization, every other methodological aspect is a straightforward replication of Lelo de Larrea-Mancera *et al.* (2020) and Lelo de Larrea-Mancera *et al.* (2022). These studies have established expectations for normal ranges of performance in each test as well as their reliability across a range of variations of environmental settings (laboratory vs home) and external noise (70 dB cafeteria noise). The only notable methodological deviations concern the inclusion of a self-reported hearing handicap questionnaire, and a wider age range to characterize young adults. Both of these variables are treated extensively in the supplementary material.^1^

Here, we collected data from a sample of 96 young adults without hearing complaints in Mexico City and compared the reliability of performance to previously published data sets from the English-version of the same PART battery (Lelo de Larrea-Mancera *et al.*, 2020; Lelo de Larrea-Mancera *et al.*, 2022). This study represents a first step towards a larger goal of collecting, and helping others collect, a wide variety of diverse and representative datasets inclusive of non-English-speakers and those who may not have access to traditional laboratory and clinical audiological facilities.

## II. METHODS

The PART app was localized to the Spanish-language and validated in a Mexican normal-hearing population. All instructions used to deliver the tests to participants were translated into Spanish, as were all software interfaces and speech stimuli. This Spanish-language validation study closely followed the methods of the studies it is validated against (Lelo de Larrea-Mancera *et al.*, 2020; Lelo de Larrea-Mancera *et al.*, 2022). These previous studies show similar hearing performance with PART tests conducted in different levels of environmental noise (Lelo de Larrea-Mancera *et al.*, 2020), or in at-home environments (Lelo de Larrea-Mancera *et al.*, 2022). Thus, the choice to collect data in an “office” environment rather than a sound booth both reflects the conditions used in the prior studies and will ensure that future projects, both clinical and research, can be done in whatever environment is available without concern of deviating from the procedure used here.

### A. Participants

Ninety-six Spanish-speaking adults between the ages of 18 and 45 (71 female, mean, *M* age = 26.4 years, standard deviation, *SD* = 7.9 years) volunteered to participate in the study. All participants self-identified as Hispanic or Latino and identified Spanish as their native language. Bilingual experience involved English in 90% of participants and was self-reported as a percentage of proficiency in their second language. This and other demographic features of the population tested are included in Table I. Participants were recruited by word-of-mouth, through the distribution of an informative flyer on central auditory processes at the Instituto Nacional de Neurología y Neurocirugía (INNNMVS) in Mexico City, and through the Psychology undergraduate career at the Universidad Nacional Autónoma de México (UNAM). All participants signed an informed consent form with procedures approved by the INNNMVS Human Research Review Board.

**TABLE I.** Demographic information of the tested sample. L2 stands for second language.

Sex	Age	HHIA	L2 proficiency	School years
74% female	*M* = 26.3	*M* = 33	*M* = 50.3%	*M* = 15.4
26% male	*SD* = 7.8	*SD* = 4.6	*SD* = 34.8%	*SD* = 4.6

The range of ages was selected to exclude any participants likely to be experiencing age-related hearing loss (see WHO, 2021). Audiometric evaluations were not conducted. However, all participants self-reported normal hearing. Self-reported hearing ability can be used as a reference standard in diagnosing hearing difficulties in conditions that better resemble real-world auditory challenges, and may better align with patients' experiences when compared to pure tone audiometry (Vermiglio *et al.*, 2018). Further, scores on the hearing handicap inventory for adults (HHIA) averaged 3.37 (*SD =* 4.63; range 0–22) and are consistent with normal hearing (Newman *et al.*, 1990). Last, the variable of age had no significant correlations (after corrections for multiple comparisons) to any of the measures collected nor their composite. Details on the HHIA scores and age, and their relationship to the rest of the measures can be found in the supplementary material.^1^

### B. Procedure

**FIG. 1.**
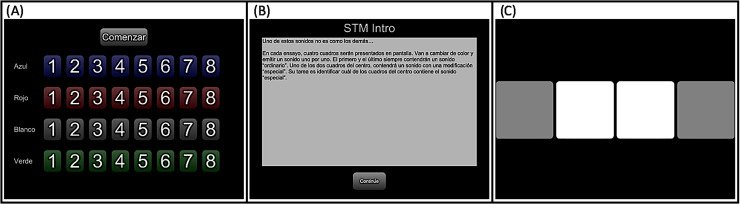
(Color online) PART example screens for the CRM response grid (A), automated instructions (B), and 4-interval 2-alternative forced choice response interface.

All procedures were carried out at the INNNMVS. After participants provided consent and answered demographic questionnaires, they were assigned to individual testing stations that consisted of a single desk with vertical dividers that separated the two stations. Automated psychophysical testing was conducted using the PART app. Instruction screens—translated to Spanish by the first author (who is proficient in English and whose native language is Spanish)—guided participants through practice runs of each test with a few easily detectable examples [see Fig. 1(B)]. Next, the program automatically proceeded to the full assessments in the progression described in the following.

Participants always began testing with a 2-kHz tone detection task, first in silence and then in competition with white noise, to familiarize them with the task structures used in PART. This familiarization phase was followed by either non-speech tests that used a 4-interval, 2-alternative forced-choice structure [see Fig. 1(C)], or the speech-on-speech masking tasks that used a response grid [see Fig. 1(A)]. Test order was counter-balanced across participants and across sessions. Participants were given small breaks between blocks of testing. After the first session was concluded, participants scheduled a second session between one day and three week after the conclusion of the first session (*mean days* = 8.5, *SD* = 6.6). The second session was identical to the first except that (1) the initial paperwork (informed consent and collection of demographic information) was only completed in the first session and (2) in the second session, participants completed a self-reported hearing handicap instrument (detailed in the following section). Each session took about 1 h to complete.

### C. Materials

Participants were tested in one of three available workstations in the lab. Although the space was generally quiet, some environmental noises were present (e.g., short quiet conversations, walking). To minimize noise, posted signs made people aware of occurrence of auditory testing. Tests were conducted using calibrated circumaural Sennheiser 280 Pro headphones (Sennheiser electronic GmbH & Co. KG, Wedemark, Germany). iPad tablets (Apple Inc., Cupertino, CA) were used to run the PART app, and were calibrated using the recommended PART calibration routine (Gallun *et al.*, 2018). In brief, equipment in PART is calibrated by measuring the headphone output for a 1-kHz tone by connecting an external microphone to a second iPad that is running a sound level meter app. PART contains a calibration module that allows the measured level in dB sound pressure level (SPL) to be input, after which PART adjusts its output to match the desired output level, which was the same for all listeners. Validation is conducted by playing a second 1-kHz tone and comparing the intended level with the measured level. The entire process is automated with simple instructions and input screens and takes only a few minutes. Further details on this procedure and the equipment used are found in Gallun *et al.* (2018) and Gallun (2020).

#### 1. Developing the Spanish-language CRM

All 256 sentences for all combinations of four colors, eight numbers, and eight callsigns of the original CRM were translated into Spanish. Original CRM sentences have the following structure: “Ready *callsign*, go to *color number* now” which was translated into Spanish as the following: “Listo *callsign*, ve al *number color* ahora.” All sentences were recorded to be 2 s in duration. The original (English) callsigns were Charlie, Ringo, Laker, Hopper, Arrow, Tiger, Eagle, Baron; the Spanish equivalents developed for this study were Carlos, Bravo, Delta, Lima, Flecha, Tigre, Sierra, Tango. These callsigns were selected to match the originals in terms of number of syllables and distribution of phonemes as closely as possible. Two callsigns (Carlos and Tigre) were literal translations of the English-version, the others were chosen to closely match the form of the English-version. The colors and numbers were literal Spanish translations of white, green, red, and blue (blanco, verde, rojo, azul) and numbers 1 through 8 (uno, dos, tres, cuatro, cinco, seis, siete, ocho).

**FIG. 2.**
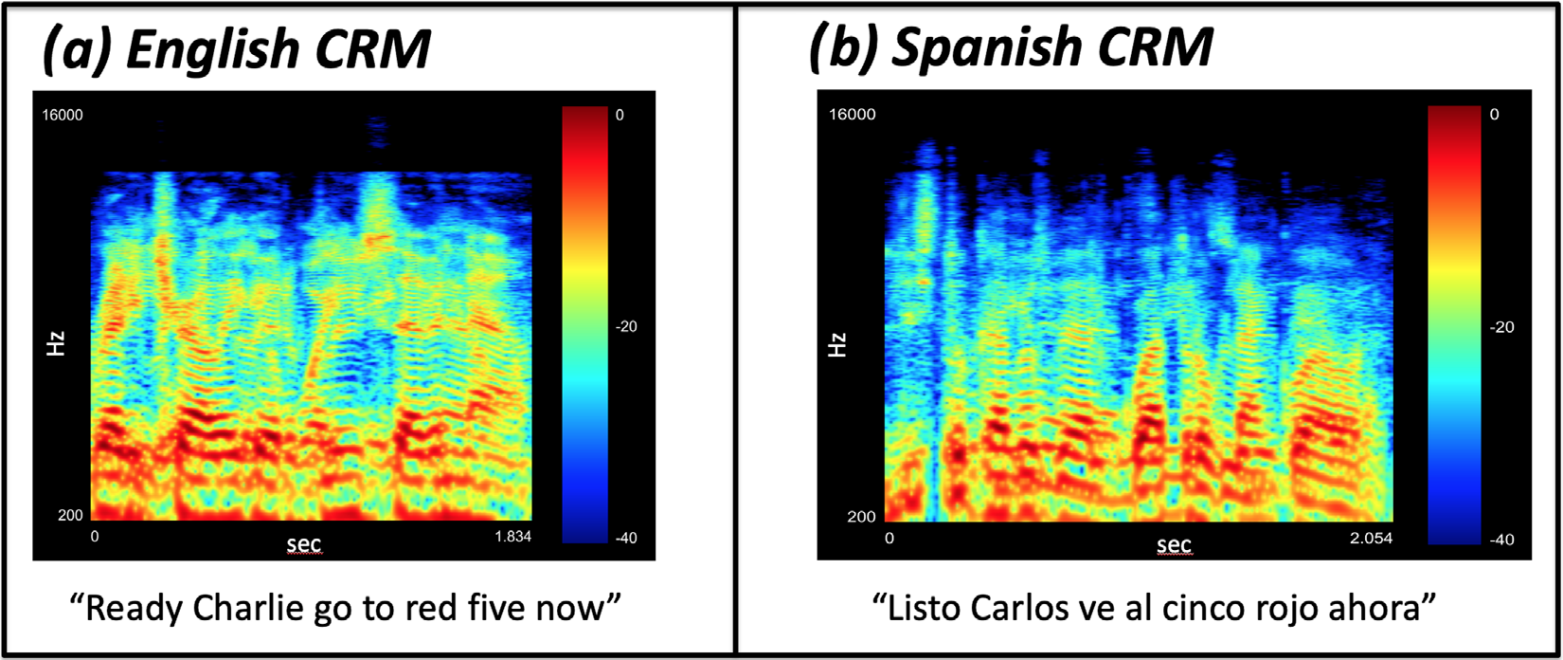
(Color online) Visual descriptions for the CRM corpus stimuli. (a) Shows a spectrogram for the English version described by Bolia *et al.* (2000). (b) Shows a spectrogram for the Spanish version developed and validated in the current study. For further analysis of the spectral and prosodic properties of the stimuli see the supplementary material (Ref. 1).

Four male and four female voices were recorded, each speaking the complete set of Spanish-language CRM sentences. For the current study, the male voices were selected for study to match previous work from our group that utilized the male voices from the CRM (Lelo de Larrea-Mancera *et al.*, 2020; Lelo de Larrea-Mancera *et al.*, 2022; Srinivasan *et al.*, 2020; Diedesch *et al.*, 2021). Stimuli were recorded in a 4 m by 2 m sound-proof booth in the Neuro-otology department of the INNNMVS using a Shure SM35-XLR head-mounted microphone connected to an inline preamplifier, and then connected through an XLR cable to a Zoom H4n recorder used as an analog-to-digital converter. The Zoom H4n was then connected with a USB cable to a Mac Air (2020) computer that used matlab (Mathworks Inc., Natick MA) to record the signal at a 44.1-kHz sampling rate with 16 bits per sample. To ensure consistent prosody and speech rates, a matlab based program first played an audio sample of the sentence that the person speaking the sentences then repeated (further detail to come). A recording period of 3.5 s was present immediately after the sample audio was played. At this point, each sentence was hand edited to ensure that each speech waveform started and ended with 0.1 s of silence and that this duration was as close to 2 s as possible. Recordings that did not match criteria were discarded. Each talker repeated each sentence until the criteria were met. The goal of this recording technique was to obtain sentences all with very similar durations, with the aim of matching the original CRM corpus sentences, which average 1.87 s in total duration. The generated Spanish-language recordings averaged 2.004 s (*SD* = 0.01) in total duration (see Fig. 2).

The first set of recordings was generated with a single talker who was fluent in English and Spanish using the original CRM audio files (in English) as the example sentences, as no Spanish-language samples were yet available. After this first set of Spanish-language recordings was complete, it was used as the Spanish-language sample for the recordings of the rest of the talkers. All the talkers used for recording the Spanish-language CRM were native Spanish-speakers. This facilitated consistent prosody across the talkers used in the corpus. Recordings were reviewed by the first author live during the recording session. The first author required each sentence to be repeated by the talkers until it was judged to have achieved the intended timings. The first author (ESLLM) and fourth author (LC) also verified that all words were spoken with equal emphasis and recordings were repeated when necessary. Total recording time for each talker was between 3 and 4 h. All recordings were post-processed to match the original CRM. Each sentence was bandpass filtered (0.08–8 kHz) using a fifth-order Butterworth filter. Overall intensity was equalized using the root mean square (rms) normalization function in matlab and the following operation: sound vector*(0.1/rms(sound vector).

### D. Assessments

#### 1. Spatial release from masking task

The tasks involved listening for a target callsign (Carlos) and reporting the color (blanco, verde, rojo, azul) and number (1–8) spoken by the talker who said that callsign at the beginning of their sentence. Responses were given on a grid showing four rows of buttons, each with numbers 1 through 8 and colored with one of the possible target colors. The target callsign (Carlos) was kept constant across trials while the target colors and numbers changed randomly on every trial. The talker who spoke the target callsign was also selected randomly on every trial. The target stimuli were presented at a fixed rms level of 65 dB SPL. Two additional talkers each spoke a masking sentence, which was selected pseudo-randomly from the remaining choices of talker, callsign, color, and number without repetition. All sentences in the corpus could be selected as target and maskers following the previously noted rules on every trial. Masker levels started at 57 dB SPL and were increased by 2 dB every two trials, eventually covering a range of 10 target-to-masker ratio (TMR) values from +8 dB to –10 dB across 20 trials. As in Gallun *et al.* (2013), TMR at threshold was estimated by the simple heuristic: number of errors, 10. This task was delivered in two different spatial conditions (spatially colocated and spatially separated), with 0-, 45-, and –45-degree spatial locations simulated over headphones. Simulations were developed with head-related-impulse-responses (HRIRs) based on the KEMAR recordings in the CIPIC database (Algazi *et al.*, 2001). HRIRs were downloaded from the Music and Audio Research Laboratory at NYU (MARL-NYU; Andreopoulou and Roginska, 2011) and the HRIRs corresponding to each of the different spatial locations were convolved with the target and masking speech (see Xie, 2013 for details), as in previous work (Gallun *et al.*, 2013; Jakien *et al.*, 2017; Gallun and Jakien, 2019; Lelo de Larrea-Mancera *et al.*, 2020; Lelo de Larrea-Mancera *et al.*, 2022). The 50% thresholds estimated from these two conditions were then used to estimate SRM.

Dependent variables were associated with the following:

(1) *Colocated condition*—The target speaker and the maskers were all presented in a center position at 0 degrees, spatially colocated in simulated auditory space.
(2) *Separated*—The two maskers were lateralized 45 degrees to the left and right of the target talker in simulated auditory space.
(3) *Spatial Release from Masking (SRM)*—The difference in estimated thresholds between the spatially colocated and separated conditions. SRM is an index of the benefit in TMR a listener obtains from the simulated spatial separation of the talkers.

#### 2. Non-speech auditory processing tasks

The non-speech auditory processing tests were used both as a further examination of the success of localizing the PART automated testing to Spanish and to examine the degree to which the auditory abilities of this sample matched those of people previously tested with the English-version of PART. These tests followed methods described by Lelo de Larrea-Mancera *et al.* (2020) with the exception that instructions were provided in Spanish. Tests used a 4-interval, 2-alternative forced-choice structure. Visually, this was presented as a horizontal row of four squares that would each illuminate briefly in sequence. While each was illuminated, a sound was simultaneously presented over the headphones. The first and last squares were always associated with a standard (or “cue”) and the second and third squares were the test intervals. One of the test intervals contained a standard and the other one contained a target sound or sound modulation specific for each test (details to follow). The target interval was selected randomly across trials. The task of the listener was to press the square that had been illuminated when they heard the target sound. An adaptive parameter specific to each task (described in the following) was changed based on participant performance following a two-stage staircase and a 2-down 1-up rule with uneven step sizes (see Levitt, 1971; García-Pérez, 2001, 2011). In this procedure, the target stimulus was made perceptually less salient (e.g., shorter gap or reduced modulation depth) following two consecutive correct responses, and more salient (longer gap or increased modulation depth) following one incorrect response. Ascending steps (making the task easier) were 1.5 times larger than descending steps (specifics for each task are detailed in the following). The first stage of the staircase had larger step sizes than the second and consisted of three reversals (changes in direction of the staircase algorithm governing target saliency) before transitioning to the second stage. The second stage consisted of six reversals, after which the adaptive track would finish. Threshold values (estimating the 77.5% correct point of the psychometric function) were estimated by taking the geometric mean of the last six reversals. Note that this method is a variation of the adaptive tracking described by Levitt (1971). It is based on the work of García-Pérez (2001, 2011), who argues that fixed step sizes lead to unstable threshold estimates. In addition, this was the method used in the comparison experiments. Each of the tasks using this algorithm took about 5 min to finish.

(1) *Temporal Gap detection task*—This task measures the smallest temporal gap that could be detected between two clicks (4 ms truncated Gaussians with an equivalent rectangular duration of 2 ms) presented at a peak equivalent level (peSPL) of 80 dB. Initial gap duration was 20 ms and gap duration was adaptively varied on an exponential scale with the first stage involving descending steps of 2^1/2^ ms and the second stage involving descending steps of 2^1/10^ ms. As mentioned previously, the ascending step size was 1.5 times the descending step size within each stage.
(2) *Frequency Modulation (FM) detection tasks*—This task measures temporal fine structure sensitivity through the detection of FM (Grose and Mamo, 2010). On each interval of the 4-interval, 2-alternative forced-choice task, a 400-ms, 75 dB SPL carrier tone was randomly drawn from a rectangular distribution between 460 and 550 Hz, and, in the target interval, the carrier tone was sinusoidally modulated in frequency at a rate of 2 Hz. The modulation started at a phase of 0 radians in both ears and modulated sinusoidally between +1π and −1π. In the diotic FM task, the modulation was applied identically to the signals presented to the two ears. In the dichotic FM task, the modulator was inverted in phase at the two ears, so that when one ear's modulator was at −1π, the other was at +1π, resulting in an interaural phase difference, which could potentially be detected as a change in the location of the carrier tone inside the head. For further details, see Palandrani *et al.* (2021). Participants were instructed to ignore the small differences in pitch height among the intervals (due to the roving of carrier frequency) and to try to detect a relatively slow “wobble.” The diotic FM task was associated with a tonal percept in the center of the head that “warbled” or “wobbled” on target intervals, while the dichotic FM task was associated with a percept that was centered in the head on standard intervals and that moved within the head (at a rate of 2 Hz) on target intervals. The modulation depth determined the amount of the wobble and the distance of the motion. The modulation depth was adapted on an exponential scale starting at 6 Hz (for example, a 500-Hz tone would modulate in frequency between 497 and 503 Hz). The first stage used descending steps of 2^1/2^ Hz and the second stage used descending steps of 2^1/10^ Hz. Again, the ascending step size was 1.5 times the descending step size within each stage.
(3) *Spectral, Temporal, and Spectrotemporal detection tasks*—These tasks measure sensitivity to broadband noise modulations in time (TM), spectrum (SM), or both (STM) (Bernstein *et al.,* 2013). Unmodulated broadband noise with a spectral range of 0.4 to 8 kHz was presented at 65 dB SPL for 500 ms. Participants were instructed to detect the broadband noise interval with a modulation present. Sinusoidal modulation was applied on a logarithmic amplitude scale (dB) and its depth was measured from the middle to the peak of the amplitude range as described in Stavropoulos *et al.* (2021). Spectral modulation (SM) was fixed at 2 cycles per octave (SM and STM) and temporal modulation (TM) at 4 Hz (TM and STM). The adaptive parameter was the modulation depth (M) dB with a starting value of 6 dB and first stage descending steps of 0.5 dB and second stage descending steps of 0.1 dB. The ascending step size was 1.5 times the descending step size within each stage.

#### 3. Self-reported hearing ability

To further characterize the study population, we collected data on self-reported hearing ability based on the hearing handicap inventory for adults (HHIA) by Newman *et al.* (1990), and the translation work of Lichtenstein and Hazuda (1998) for a Mexican-American population. The authors made minor adjustments to the wording for readability. The version used in this study can be obtained from the supplementary material.^1^ We scored the instrument without dividing it into sub-scales as there is no evidence that the scales originally described have discriminant validity (Cassarly *et al.*, 2018). Participants were instructed to respond to each of 25 items related to hearing problems on a scale ranging from “no” (0 points), “a veces” (sometimes; 2 points), or “sí” (yes; 4 points). The total score ranges from 0 to 100, with higher scores indicating increasing levels of self-perceived hearing handicap.

### E. Statistical analysis

To examine the degree to which the Spanish-language and English-language speech tests resulted in similar ranges of estimated thresholds, we compared the thresholds obtained in this study with those obtained by Lelo de Larrea-Mancera *et al.* (2020) and Lelo de Larrea-Mancera *et al.* (2022). These datasets collectively include a range of at-home and in-lab environments (Lelo de Larrea-Mancera *et al.*, 2022), including in-lab under moderate recorded cafeteria noise levels of 70 dB SPL (Lelo de Larrea-Mancera *et al.*, 2020). To facilitate the analysis for the present study, we combined the two previous datasets. This approach was appropriate because the difference in the range of thresholds in the two studies was shown to be within the measurement error of the tests (Lelo de Larrea-Mancera *et al.*, 2022). The same outlier rejection criterion was employed, which involved removing datapoints that exceeded three standard deviations from the mean of the distribution formed from the combined datasets of Lelo de Larrea-Mancera *et al.* (2020) and Larrea-Mancera *et al.* (2022).

To assess the difference between the measures in this study and the previous English-language versions, we examined both test-retest correlations and limits of agreement. The similarity of test-retest correlation across studies was formally compared with a Fisher *z* test as specified in Meng *et al.* (1992) and corrected for multiple comparisons as specified in Holm (1979). A non-significant *z*-test indicates similar reliability of measures across datasets. One drawback of testing correlations is that they are negatively impacted by restricted ranges of between subject variability and that they are not sensitive to a fixed change in threshold applied to all the data. For this reason, we also calculated the limits of agreement, which indicate the size of the within subject variability across sessions without being affected by the between subject variability (Bland and Altman, 1999), and t-tests to reveal any fixed shifts in the collected measures. All statistical analyses and figures were conducted in matlab (Mathworks Inc., Natick MA), and JASP (JASP Team, 2022).

## III. RESULTS

Results are divided into two sections for the purpose of clarity. First, in Sec. III A, we provide results of the Spanish-version of the SRM task. Then in Sec. III B, we provide results for the non-speech measures.

### A. Does the Spanish-language CRM provide reliable measures of spatial release from masking?

**FIG. 3.**
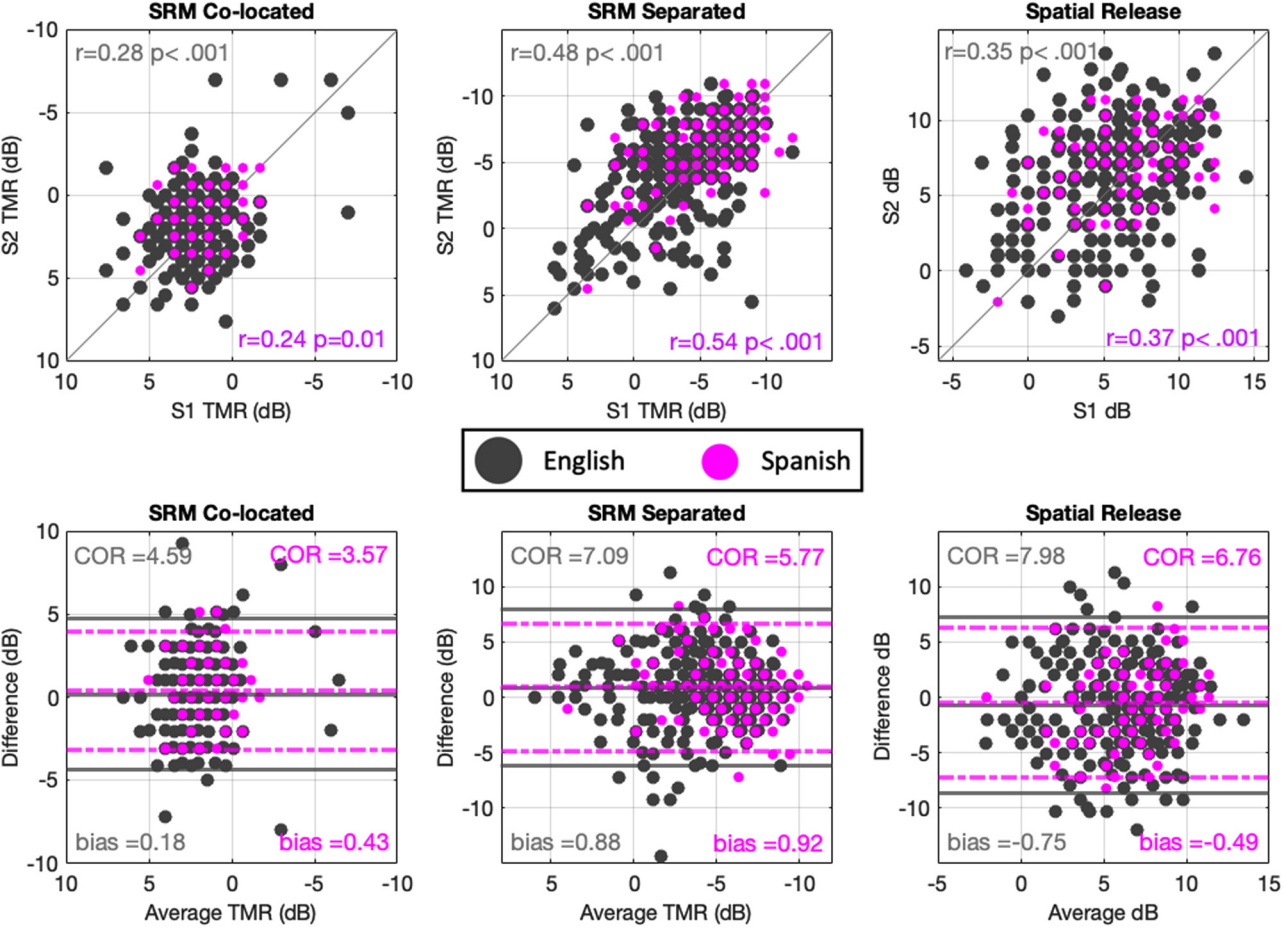
(Color online) Scatter plots showing performance on both sessions of the spatial release from masking task in its spatially colocated and separated conditions plus the spatial release metric in this study compared to the combined datasets of of Lelo de Larrea-Mancera *et al.* (2020) and Lelo de Larrea-Mancera *et al.* (2022). Panels on the top show correlations across sessions (all significant *p* < 0.01 except SRM task Colocated condition for Spanish *p* = 0.01). Bottom panels show limits of agreement between sessions. Axes were inverted for the colocated and separated TMR values, so that better performance is oriented towards the top (top row) and the right (bottom row).

To assess the reliability of the Spanish-language spatial release from masking test, we compared the correlations and limits of agreement of the measures across two sessions for these data and for the data collected with the English-language version (see Fig. 3, top row). We found significant correlations (all *p* values *≤* 0.01) for all test-retest data, as was the case with the English-version (also plotted in Fig. 3). We also found the correlations had similar magnitudes (differences of 0.02–0.06) for the English and Spanish tests. A Fisher's *z*-test confirmed that the correlations were not significantly different across datasets (Colocated *z =* –0.36, *p =* 0.72; Separated *z =* 0.69, *p =* 0.49; SRM *z =* 0.19, *p =* 0.84) indicating similar test-retest reliability.

The limits of agreement are a metric used to compare the average threshold across both sessions for each participant (plotted on the *x* axis of the plots in the bottom row of Fig. 3) with the difference between sessions for each participant (plotted on the *y* axis of the plots on the bottom row of Fig. 3). The differences across sessions can be used to assess test-retest reliability, and the relationship with average threshold can be used to determine whether there is better reliability for better or worse performers. This method also allows visual inspection of between-subject variability in the horizontal and within-subject variability in the vertical orientation. Further, it is robust to data aspects such as restricted ranges of between-subject variability, which is known to impact correlations (see Bland and Altman, 1999). A coefficient of reliability (COR) can be extracted from the standard deviation of the differences across session multiplied by the critical value of 1.96. This COR is set above and below the mean differences across sessions as the limits of agreement (see Fig. 3). Also, a bias metric indicating mean systematic differences favoring one session or the other is provided. Limits of agreement for both the English and Spanish versions are shown in the bottom row of Fig. 3. The bias between the datasets is very similar, but the COR indicates a reduced parametric region for expected differences between repeated measures in the Spanish test as compared to the English-version.

Last, a series of independent samples t-tests explored whether estimated thresholds were significantly different on average between the previously collected English-language datasets and the present Spanish-language dataset. In addition to the usually reported *t* and *p* statistics, we also report Cohen's d as a measure of effect size (expressed in units of standard deviation), and Bayes factor 10 (*BF_10_*) as a continuous estimate of the amount of evidence in support of the null hypothesis (*BF_10_ <* 1), or in support of the alternative (BF_10_ > 1) (Wagenmakers *et al.*, 2018). As can be observed in Fig. 3, the Spanish test led to slightly better performance (lower thresholds) than was observed in the previously collected English datasets. The Spanish-language SRM task in the spatially colocated condition had statistically significant lower thresholds, with a mean difference of 0.99 dB (*t_(368)_ =* 6.14, *p <* 0.001, *Cohen's d =* 0.72, *BF_10_ >* 4 318 000), also the separated condition had statistically significant lower thresholds, with a mean difference of 1.81 dB (*t_(372)_ =* 4.94, *p <* 0.001, *Cohen's d =* 0.58, *BF_10_ =* 11 443.4). The spatial release metric was greater for the Spanish dataset, with a statistically significant mean difference of 1.14 dB (*t_(368)_ =* 3.07, *p =* 0.003, *Cohen's d =* –0.39, *BF_10_ =* 22.6). These t-test analyses were corrected for multiple comparisons (Holm-Bonferroni).

### B. Characterizing performance on non-speech tests

**FIG. 4.**
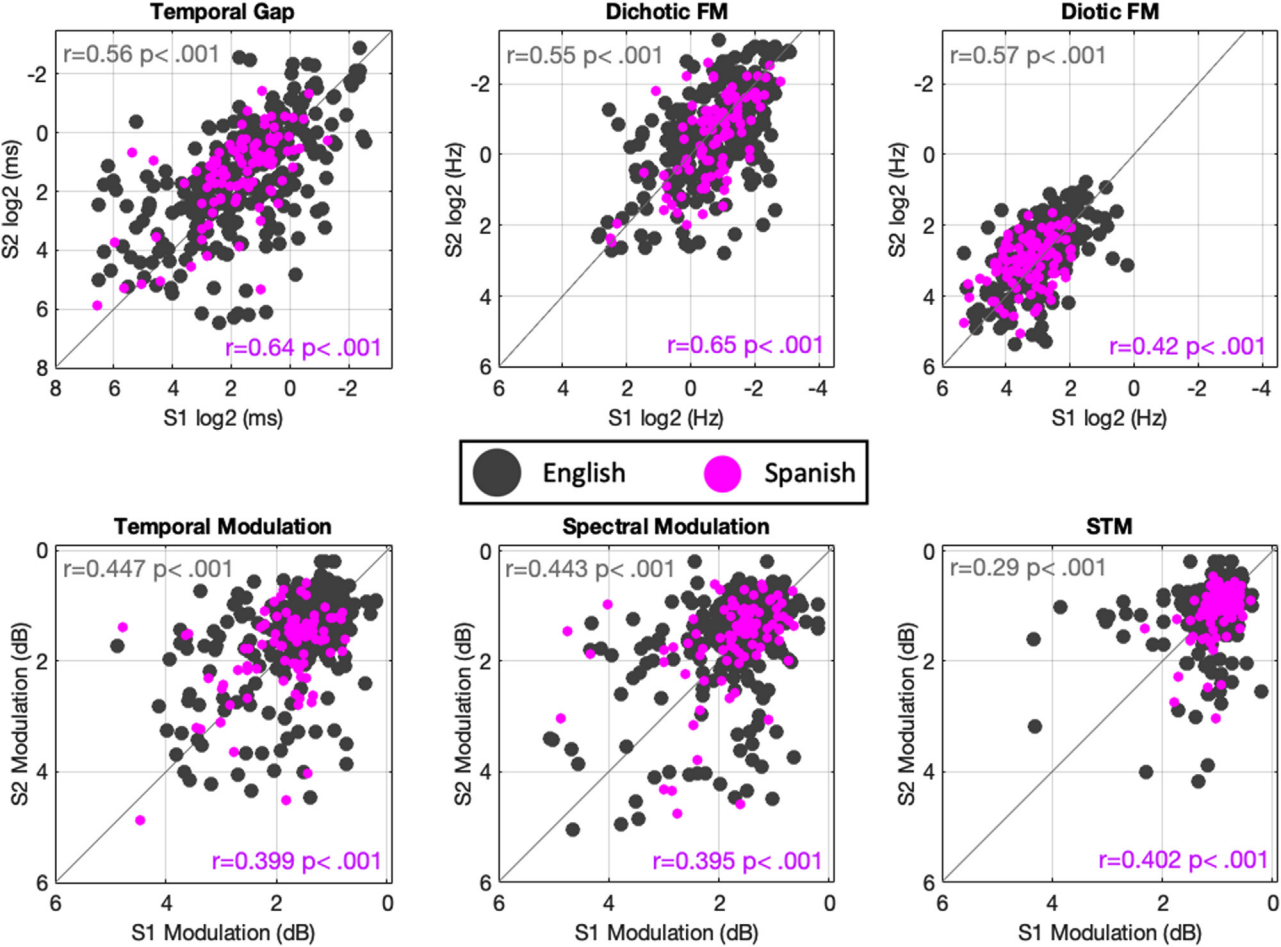
(Color online) Scatter plots comparing performance across two sessions (test-retest) in non-speech auditory processing measures of this study in contrast to the merged datasets of Lelo de Larrea-Mancera *et al.* (2020) and Lelo de Larrea-Mancera *et al.* (2022).

To characterize performance on the non-speech measures of auditory processing, we compared the data collected in this study to these measures in the same two previously published datasets (Lelo de Larrea-Mancera *et al.*, 2020; Lelo de Larrea-Mancera *et al.*, 2022). Descriptive data for each task across both this study and the combined English-language studies are provided in Table II. Overall distributions of performance in this study were similar to those reported in the previous data sets (see Fig. 4). As with the speech tests, test-retest correlations between the first and second test sessions were similar for this data set and for the previous data sets. There was no systematic increase or reduction in correlations found for the current study as compared with previous data. The differences were examined statistically with Fisher's z-tests and no significant results were obtained, either before or after correction for multiple comparisons.

**TABLE II.** Descriptive statistics for the average thresholds across sessions for the speech and non-speech tasks. Data for the Gap, Dichotic, and Diotic FM were transformed to logarithmic units for analysis and were transformed back to ms and Hz for display purpose.

	Group	Valid(n)	Unit	Mean	Median	SD	SE	Range	Min	Max
Gap	US datasets	280	ms	5.96	3.08	7.85	0.47	54.48	0.16	54.64
	MEX dataset	96	ms	5.41	2.55	9.81	1.002	73.44	0.51	73.95
TM	US datasets	276	(M) dB	1.92	1.5	1.22	0.07	6.9	0.55	7.45
	MEX dataset	92	(M) dB	1.91	1.76	0.72	0.07	3.7	0.96	4.66
SM	US datasets	278	(M) dB	1.97	1.56	1.16	0.07	7.05	0.59	7.64
	MEX dataset	94	(M) dB	1.68	1.47	0.69	0.07	3.25	0.7	3.95
STM	US datasets	272	(M) dB	1.39	1.01	1.03	0.06	7.58	0.46	8.05
	MEX dataset	96	(M) dB	1.06	0.97	0.44	0.04	3.51	0.6	4.11
Dichotic FM	US datasets	278	Hz	1.25	0.58	2.21	0.13	25.74	0.12	25.87
	MEX dataset	95	Hz	0.89	0.6	0.91	0.09	5.32	0.17	5.5
Dichotic FM	US datasets	275	Hz	10.42	7.25	21.52	1.29	347.2	1.88	349.09
	MEX dataset	95	Hz	9.96	8.88	4.9	0.5	28.7	4.005	32.78
Colocated	US datasets	274	TMR dB	2.28	2.45	1.42	0.08	11.58	−5	6.58
	MEX dataset	96	TMR dB	1.29	0.9	1.16	0.11	6.7	−1.67	5.03
Separated	US datasets	278	TMR dB	−3.86	−4.25	3.23	0.19	16.43	−9.93	6.5
	MEX dataset	96	TMR dB	−5.67	−5.8	2.68	0.27	14.45	−10.45	4
Spatial Rel.	US datasets	280	Db	5.82	6.19	3.05	0.18	17.54	−2.06	15.48
	MEX dataset	96	dB	6.96	7.74	2.51	0.25	13.41	−2.06	11.35

The limits of agreement analysis (see supplementary materiary^1^) also matched the data from the speech tests, with slightly better test-retest reliability for the data obtained in this study. Overall, these data show that Spanish PART has good test-retest reliability across sessions that are similar to the English-version and that participants in this study had auditory processing thresholds within the range previously reported for English-speaking undergraduates (mean age of 19) with no reported hearing difficulties. An exploratory set of mean comparison analyses between the English and Spanish datasets is provided in the supplementary material (see supplementary material, Table S2^1^). This can be used to further corroborate that the hearing abilities of the participants in both datasets are comparable.

## IV. DISCUSSION

This study demonstrated that a novel Spanish-language version of the CRM corpus (see Bolia *et al.*, 2000) obtained thresholds on a SRM task (Gallun *et al.*, 2013) that closely match previous data sets collected using the original (English) CRM sentences and the English-language version of the PART app (Lelo de Larrea-Mancera *et al.*, 2020; Lelo de Larrea-Mancera *et al.*, 2022). While some small differences in performance may exist between these datasets (see the following discussion), overall, these data demonstrate that it is feasible to collect reliable data with the new Spanish-language CRM corpus and six other auditory processing tests using the new Spanish version of PART.

We found that participants' performance was slightly better in the Spanish version of the SRM task when compared to the previously published results from the English version of the SRM task. The fact that this occurred primarily in the separated condition (1.85 dB *mean difference*) leads to greater differences between colocated and separated, and thus greater SRM. It is unclear at this point whether this difference is related to (1) differences in auditory processing between the populations sampled; (2) differences in the stimuli recordings made; or (3) differences in the intelligibility of the languages employed. The first seems unlikely due to the similarity in the non-speech measures between this and the previous studies. The second is addressed in the supplementary material,^1^ where it is shown that there are potential differences in the prosodic information between the two corpuses as revealed by the modulation spectra. In the future, it is perhaps the case that further analysis of the spectrotemporal characteristics of the different speakers present in both the Spanish and the English versions of the CRM may clarify this point.

The third possibility, that the languages are differentially intelligible in this task, could be consistent with small differences in performance observed in other studies, where Spanish-language performance is better than English-language performance (Lew and Jerger, 1991; Von Hapsburg and Peña, 2002). This difference has been mentioned as a potential source of concern for validity of the assessments in English for non-native English-speakers (Cooke *et al.*, 2008; Hisagi *et al.*, 2022). One possible explanation for this difference in this and other studies is there are fewer vowel sounds in the Spanish language than in the English language. In addition, there is one-to-one phoneme-to-grapheme correspondence in Spanish, which is not the case in English. While particularly salient, it should be noted that these are just a few of the many differences between English and Spanish that could have led to the improved performance. Further, it is not clear why these differences would be more evident in the spatially separated than the colocated condition, although this may just reflect the wider range of thresholds obtained in the separated condition for both data sets. Specifically, while the difference was almost twice as great in the spatially separated than in the colocated, the standard deviations were also roughly twice as large. In addition, the much larger sample size of the combined English data sets could have led to a greater number of participants with either very large or very small thresholds. It is worth noting that such differences were not found with the non-speech tests, however. As mentioned previously, that a difference was found in the speech tests but not in the non-speech tests suggests that the difference may be specific to speech testing, which would be an interesting direction for future work, should it be replicable.

Another possibility for why the difference in the number of very large or very small thresholds between the English and Spanish datasets was only found for the speech stimuli is that the “informational masking” (IM) (Durlach *et al.*, 2003) of the English-language CRM may be greater than that of the Spanish-language CRM. It is commonly found that increasing IM leads to increased inter-subject variability. This is also consistent with the idea that some aspects of the Spanish language may lead to less similarity and thus greater intelligibility of the Spanish keywords as compared with the English keywords. Further, and of the greatest interest in regard to understanding hearing health needs, data from participants with hearing difficulties are needed to establish the validity of these tests for their use in different areas of basic and clinical research. We note that the primary contribution of the current manuscript is a demonstration of the reliability of the Spanish-language measures and their feasibility of use as a tool to reach populations that are historically understudied in terms of basic and clinical research and underserved in terms of hearing healthcare.

While previous studies (Lelo de Larrea-Mancera *et al.*, 2020; Lelo de Larrea-Mancera *et al.*, 2022) reported data from undergraduate students around 19 years of age (*M =* 19.6, *SD =* 2.31, *range* = [18–30]), here we collected a sample with an extended age range (*M =* 26.4, *SD* = 7.9, *range* = [18–45]). The broader age range used in this sample could theoretically have been associated with some of the small differences in thresholds observed. However, we found no association between age and performance on any of the tested measures in this sample (correlations of age to each test in each session are provided in the supplementary materials). We included an extended range of ages because the definition of a young adult is ambiguously defined, and world trends of hearing suggest age effects are to be found after the age of 50 (see WHO, 2021). Further, we note that sampling disproportionately from populations of younger listeners with no hearing complaints is largely an artifact of testing populations of convenience and in many cases does not reflect meaningful design choices. In this study, we intentionally recruited participants who reported having no hearing difficulties, which was a design choice that was made to better match the previous data but that may have biased our sample from the “normal” aging population within the studied range. This may have obscured some of the potential relationships with age that might have been observed otherwise, such as those noted by Gallun *et al.* (2013), who did not exclude based on age or hearing difficulties. Future research across a broader age range, including those with hearing difficulties, will be required to better capture the effects of age on the general population.

The current study was conducted in Mexico City, Mexico. While the current study's Spanish localization of the PART system potentially makes these measures of central auditory processing accessible to Spanish speakers worldwide, future research will be required to investigate if variations exist among Spanish speakers in other parts of Mexico, other Spanish-speaking countries, as well as Spanish-bilinguals. This includes Spanish speakers in the US, all of whom represent populations who are currently not well-served by existing psychoacoustic measures. While this can be considered a limitation of the current study, we note that this is also a limitation of the English-language versions of the CRM in general and most other hearing tests that are rarely localized to other languages, or validated across the diverse speakers who may be the target of such tests. Thus, while more research may be required, the availability of portable, automated Spanish-language auditory processing assessments will allow clinicians and researchers to begin to collect data among minoritized and historically underrepresented groups.

In conclusion, we provide here a validated Spanish-language version of the CRM (see Bolia *et al.*, 2000) in a SRM task, and reproduce ranges of performance values across several auditory processing measures delivered in the Spanish language to a population of young listeners without hearing complaints in Mexico City. The Spanish-language corpus, along with the other auditory processing tests used here, is available in PART online for free download and use. PART can be downloaded for different operating systems across several electronic platforms (see https://ucrbraingamecenter.github.io/PART_Utilities/).

PART has many features and attributes that can now be used to support basic research and has the potential to provide a clinical evaluation of individuals with restricted access to clinical facilities. In addition, PART has the potential to be included in research studies, and bring hearing healthcare to populations that have been historically underserved, such as those in rural communities. The key features that support this future are its availability on portable platforms and the finding, supported by the data reported here, of consistent ranges of performance across multiple testing environments, such as between an in-lab and an at-home setting (see Lelo de Larrea-Mancera *et al.*, 2022). The data reported here provide a framework that we believe can be replicated to address the hearing needs of other populations of English and Spanish-speakers as well as speakers of many other languages around the world. We thus believe that the current study represents an important step towards more inclusive and representative research that truly aims to address the hearing needs of our world's populations.

## Footnotes

^1^
See supplementary material at https://www.scitation.org/doi/suppl/10.1121/10.0016850 for additional analysis on the reported datasets and stimuli used.
